# Nicotinamide mononucleotide (NMN) supplementation ameliorates the impact of maternal obesity in mice: comparison with exercise

**DOI:** 10.1038/s41598-017-14866-z

**Published:** 2017-11-08

**Authors:** Golam Mezbah Uddin, Neil A. Youngson, Bronte M. Doyle, David A. Sinclair, Margaret J. Morris

**Affiliations:** 1 Department of Pharmacology, School of Medical Sciences, UNSW Sydney, Sydney, NSW-2032 Australia; 2 Department of Genetics, Paul F. Glenn Center for the Biology of Aging, Harvard Medical School, Boston, MA-02115 USA

## Abstract

Maternal overnutrition increases the risk of long-term metabolic dysfunction in offspring. Exercise improves metabolism partly by upregulating mitochondrial biogenesis or function, via increased levels of nicotinamide adenine dinucleotide (NAD^+^). We have shown that the NAD^+^ precursor, nicotinamide mononucleotide (NMN) can reverse some of the negative consequences of high fat diet (HFD) consumption. To investigate whether NMN can impact developmentally-set metabolic deficits, we compared treadmill exercise and NMN injection in offspring of obese mothers. Five week old lean and obese female C57BL6/J mice were mated with chow fed males. Female offspring weaned onto HFD were given treadmill exercise for 9 weeks, or NMN injection daily for 18 days. Maternal obesity programmed increased adiposity and liver triglycerides, with decreased glucose tolerance, liver NAD^+^ levels and citrate synthase activity in offspring. Both interventions reduced adiposity, and showed a modest improvement in glucose tolerance and improved markers of mitochondrial function. NMN appeared to have stronger effects on liver fat catabolism (*Hadh*) and synthesis (*Fasn*) than exercise. The interventions appeared to exert the most global benefit in mice that were most metabolically challenged (HFD-consuming offspring of obese mothers). This work encourages further study to confirm the suitability of NMN for use in reversing metabolic dysfunction linked to programming by maternal obesity.

## Introduction

Over the last three decades, globally, the proportion of adults with a body mass index of >25 has increased to approximately 37% and 38% in men and women respectively^1^. It has also been reported that females are more likely to be obese or overweight than males^2^. Epidemiological studies have revealed associations between increased maternal obesity and increased infant birth weight^3^, adiposity^4^, and increased risk of offspring developing obesity in later life^5^. There are multiple explanations for these associations such as the shared environment (e.g. diet) and genetics of parent and child^6^, however, a key contributor is the alteration of developmental and metabolic processes in offspring in early life through ‘developmental’ or ‘gestational programming’^6–8^. These alterations in glucose and fat metabolism, and appetite regulation can predispose offspring to obesity in adolescence or adulthood^6–8^. Childhood obesity itself has been shown to increase the risk for development of later life metabolic disorders including type 2 diabetes, cardiovascular diseases, hypertension and non-alcoholic fatty liver disease^9,10^. The process by which maternal obesity raises the risk for female offspring becoming obese, in turn perpetuating the risk to the next generation, has become known as the intergenerational cycle of obesity^11^. To reduce the risk for future generations, investigating interventions that can break the cycle in females are especially important.

Due to the dramatic increase in obesity in children, prevention of childhood obesity is becoming a principal target^12^. In animal models recent studies have shown that lifestyle interventions involving dieting, exercise or the combination of both in the mother can successfully reduce body weight^13,14^, triglyceride accumulation, glucose intolerance and insulin resistance^15,16^ in offspring. Furthermore exercise during gestation can protect against hepatic steatosis and glucose intolerance in offspring of obese mothers^14,17^. Data from studies investigating pharmacological therapies in obese mothers, including conjugated linoleic acid^18^, metformin^19^, and resveratrol^20^ has shown quite successful outcomes for offspring. However, as interventions in the mother can be problematic, there is still a need for interventions in the offspring themselves. In this regard our lab previously showed that the negative metabolic impacts of maternal obesity could be partially rescued by late onset (11 week of age) exercise, or a healthy diet, in female rat offspring^21^.

Mitochondria as central mediators of cellular energy metabolism, play a critical role in the pathophysiology of obesity, and in the drug or exercise interventions mentioned above. Mitochondrial dysfunction and low energy (low ATP) production are found in obesity-related diseases such as insulin resistance and type 2 diabetes (T2D)^22–25^. In obesity and T2D, nicotinamide adenine dinucleotide (NAD^+^), an essential metabolic cofactor, is reduced^26–28^. Interconversion of NAD^+^ to NADH in the TCA cycle, β-oxidation and oxidative phosphorylation is crucial for mitochondrial metabolism and with age, NAD^+^ levels can decline by up to 50%^29^.

In animals, physical activity has been shown to improve mitochondrial biogenesis and function as well as increase the levels of NAD^+^ in primary metabolic organs^30–32^. Indeed, it is thought that many of the benefits of exercise and of fasting stem from increasing NAD^+^, which stimulates mitochondrial function leading to burning of fat and carbohydrate stores^33,34^.

In mammals, NAD is synthesised from tryptophan or recycled from nicotinamide via the salvage pathway through an intermediate nicotinamide mononucleotide (NMN). The NAD^+^ precursors, NMN and NR, are therapeutic targets for treating metabolic disorders and aging^35^. Studies including our own on mice have shown that NMN supplementation increases NAD^+^ levels, improves glucose clearance in HFD induced obesity, aging and diabetes^26,27,33^. However, while there is extensive overlap in the ultimate physiological effects of diet-induced and developmentally-programmed metabolic disease there are significant differences. Diet-induced obesity is related to increased ingestion of energy in childhood or adulthood. In contrast, effects of maternal obesity such as adiposity^4^ are evident at birth and involve alterations to oocyte state^36,37^ and the gestational milieu^7^. Therefore, while previous studies by us and others have shown the ability of NMN-supplementation to reduce liver fat content in mouse models of diet-induced obesity, it is unknown whether this intervention is effective at reversing metabolic deficits that are initiated prior to birth. We hypothesised that the benefits of NMN would be particularly pronounced in offspring liver, and would be linked to changes in fat metabolism.

Here we aimed to test whether NMN administration could ameliorate the effects of maternal obesity in female offspring consuming either regular or HFD post-weaning. The other major goal of this experiment was to contrast the effects of exercise and NMN in these female offspring. To do so, we examined effects on weight gain, adiposity and glucose tolerance, and measured mitochondrial DNA copy number as a marker of mitochondrial number, triglyceride as a marker of fat storage in the liver, and liver NAD^+^ and NADH levels. As a mitochondrial functional marker we chose the widely utilised citrate synthase activity. We also investigated the mRNA level of fat metabolism markers in liver.

## Methods and Materials

### Animal procedures

All animal experimental protocols were approved by the Animal Ethics Committee, UNSW, and carried out according to their guidelines and regulations (ethics number 13/25B). Three week old C56BL6/J female mice (n = 128, Animal Resources Centre, Perth) were housed at the Biological Resources Centre facility, UNSW, Australia. Mice were acclimatised for a week on standard chow diet. Then groups of mice of similar average body weight were assigned to either control chow (n = 48) or HFD (n = 80). HFD pellets were a semi-pure formulation for laboratory rats and mice based on Research Diets D12451 from Specialty Feeds, Glen Forrest, WA, Australia (23.5% of total weight is fat and 19MJ/kg digestible energy, SF 04-001). After 5 weeks on the different diets, 1 male adult chow-fed mouse was introduced to each cage of 4 females for 7 days. There was no significant difference in body weights of stud mice between dietary groups. Fourteen days after mating pregnant mice were housed individually; prepregnancy diet was continued throughout pregnancy and lactation. Pups were born over a 3 day period, and were left undisturbed for the first week to prevent maternal stress. At PND 24 female offspring were weaned, and distributed across chow or HFD groups, ensuring similar starting body weights. Mothers were sacrificed and tissue collected from metabolic organs after weaning. The experimental timeline is presented in Supplementary Fig. S1.

After 6 weeks of post-weaning diet, offspring from HFD fed mothers were further distributed into either sedentary, exercise, or NMN groups thus generating 6 groups: HFD-Chow-sedentary HCS; HFD-Chow-exercise HCX; HFD-Chow-NMN HCN; HFD-HFD-sedentary HHS; HFD-HFD-exercise HHX; HFD-HFD-NMN HHN (Supplementary Fig. S1). Offspring from chow mothers remained in their chow or HFD groups: Chow-Chow-sedentary: CCS; Chow-HFD-sedentary: CHS. At 9 weeks of age, treadmill running began in those offspring of obese mothers assigned to exercise as described below, and from 18 days before cull NMN or vehicle was provided.

After 5 days of training, HCX and HHX mice underwent treadmill (Columbus Instruments Exer 3/6 (0257-901 M)) running 6 days (55 min/day) per week for 9 weeks. In each session the exercised mice had a warm-up period of increasing running speed from 6 to 12 m/min. At 9 min the speed was increased to 15 m/min. In each session after 400 m, the speed was slowed to 6 m/min for 5 minutes then returned to 15 m/min for another 20 minutes. Exercise was carried out an hour before the end of the light period (1700–1830 hrs). All mice in the non-exercise groups experienced the treadmill with the belt turned off, 5 days a week. NMN was dissolved in PBS and injected i.p. daily for 18 days before sacrifice, 500 mg/kg body weight^26,27^. All non-NMN treated mice received a vehicle i.p. injection of PBS daily at the end of the light period.

### Glucose tolerance test (GTT)

At 17 weeks of age mice were fasted for 5 hours prior to the GTT. Baseline blood glucose was measured by tail nick at time 0. Mice were then challenged with an i.p. glucose bolus (2 g/kg body weight). Blood glucose was measured using Accu-ChekH glucose meter (Roche Diagnostics).

### Sample collection, processing and plasma insulin concentration

At 19 weeks NMN or vehicle was injected 4 hours before anaesthetic. Mice were culled starting at 1200 h the next day and the exercise groups had their final session the day before cull. Animals were weighed and fasted for 5 hours then mice were anesthetized (ketamine/xylazine 200/20 mg/kg, i.p.). A blood sample was collected by cardiac puncture and plasma collected after centrifugation (2000 g 10 mins). Plasma insulin concentrations were measured by Ultra-Sensitive Mouse Insulin ELISA kit (Crystal Chem Inc.). After decapitation, white adipose tissue (gonadal, retroperitoneal, inguinal), muscle (quadriceps, tibialis, soleus) and liver were weighed. Liver was ground using a tissue pulverizer (Bessman) on dry ice and liquid N_2_.

### Hepatic triglyceride, citrate synthase activity and NAD^+^-NADH assays

Triglyceride measurement was conducted as described previously^26^. Briefly, after homogenisation and extraction, liver triglyceride content was determined using a commercially available colorimetric assay kit, TG reagent Triglyceride GPO-PAP (cat# 11730711 216) from Roche/Hitachi. After incubation samples were read on a BioRad iMark plate reader.

Citrate synthase activity was assayed by homogenizing 35 mg of powdered liver in tris buffer and subjecting to three freeze-thaw cycles with liquid N_2_^26^. Data were standardised for protein content, quantified using Bovine Serum Albumin (BSA) as standard (ThermoFisher Scientific Cat#23208)

Levels of NAD^+^ and its reduced form NADH were measured as described^26,38^. Homogenised samples were extracted for protein and NAD measurements. Total NAD and NAD^+^ were quantified using a Bio-Rad Imark microplate reader; data are presented as picomoles of NAD^+^ or NADH per mg of protein.

### Mitochondrial DNA copy number and quantitative RT-PCR

Mitochondrial DNA copy number was measured by qPCR as previousy described^26^. DNA was extracted from 25–30 mg of ground liver. SYBR green Qpcr (SensiFAST SYBR, Bioline) was used to determine mitochondrial DNA copy number. Two primers were used, 36B4 for the nuclear genome (amplifies a region of the *Rplp0* gene) and *Cytb* for the mitochondrial genome^26^.

RNA was extracted using Tri-reagent (Sigma, St. Louis, MO, USA), and quantified by Biospec-nano spectrophotometer (Shimadzu Biotech, Nakagyo-ku, Kyoto, Japan). After TRI-reagent extraction ~2 μG of RNA from each sample was visualised on an agarose gel. Only samples with clear 18 S and 28 S rRNA bands (thus confirming that the RNA had minimal degradation) were used for subsequent RT-qPCR analysis.

Expression of target genes was measured using a Roche LightCycler480. Standard curves were produced for each gene using templates generated by serial dilution of a combined cDNA sample from all samples.

All sample PCRs were done in duplicate, and all genes of interest were normalized by dividing by the geometric mean of two control genes *Gapdh* and *Ywhaz*. No difference in expression of housekeeper genes was observed across treatment groups. All primer sequences are shown in Supplementary Table S4. qPCR relative fold changes between groups are presented with the CCS group given the value 1.

### Statistical analysis

Data are expressed as mean ± SEM. Body weight and GTT were analysed by 3-way mixed ANOVA. Body weight and GTT of offspring of obese mother were separately analysed based on post-weaning diet using repeated one-way ANOVA. Due to logistical constraints the design necessitated separate 2-way ANOVA analyses for all other parameters. Thus the effect of maternal and post-weaning diets using the CCS, CHS, HCS and HHS groups, and the effect of the interventions and post-weaning diets using the HCS, HCX, HCN, HHS, HHX and HHN groups used separate 2-way ANOVAs. If data were not normally distributed they were log transformed to achieve normality before analyse. Significant differences at P < 0.05 level between diet groups are shown as ^†^ overall maternal HFD effect; * overall post-weaning HFD effect. For interventions, ^#^ overall intervention effect; ^X^ simple main effect of exercise; ^ simple main effect of NMN.

## Results

### Effect of HFD on dams and mating outcome

At four weeks of age, female mice with an average body weight of 13.11 ± 0.09 g were separated into either chow or HFD groups. A week later mice fed with chow were 14.45 ± 0.18 g whereas HFD fed mice weighed 16.54 ± 0.14 g. At nine weeks of age, before mating, HFD mice were 20% heavier than chow (21.22 ± 0.37 versus 17.58 ± 0.25 g, P < 0.001). Pregnancy rates were similar in chow and HFD-fed females. There were no differences in litter size or offspring sex ratio between the dietary groups (data not shown). At weaning offspring were allocated to different diet groups, ensuring no significant differences in average starting body weights. All groups were sourced from 9–11 different mothers.

To characterise the effects of the HFD on the mothers we sacrificed 16 mice (8 from each diet group) a week after the offspring were weaned. Examination of tissue weights showed significant increases in heart, quadriceps muscle and gonadal fat mass (Supplementary Table S2). After normalisation as % body weight, we found significantly higher gonadal fat accumulation in HFD fed mothers compared to chow fed mice. As expected the basal glucose concentration was significantly higher compared to chow fed mothers (Supplementary Table S2 **)**.

### Diet and intervention effects on body weight of female offspring

**Figure 1:**
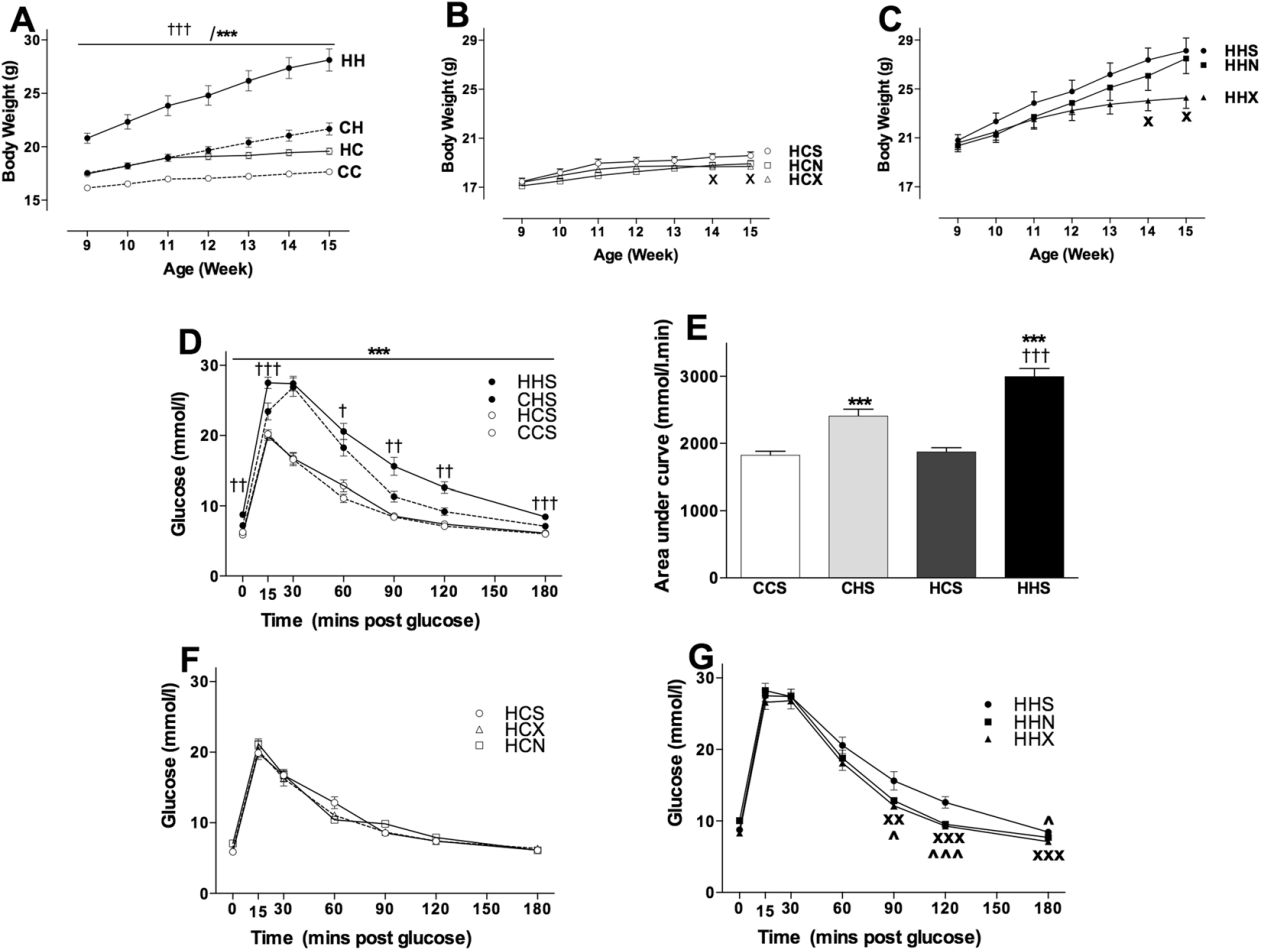
Diet and intervention effects on body weight and GTT of female offspring. Data are shown as mean ± SEM, n = 11–16 per group. The first letter represents the maternal diet and the second letter post-weaning diet; chow (**C**) or HFD (**H**); the third letter represents intervention; sedentary (S), exercise (X) or NMN (N). Abbreviations: GTT, Glucose tolerance test; AUC, Area under curve. To explore the impact of maternal and post-weaning diet on body weight and GTT in the sedentary offspring groups CCS (open circle dotted line), CHS (closed circle dotted line), HCS (open circle solid line) and HHS (closed circle solid line) were analysed by three-way mixed ANOVA (**A**,**D**). To explore the impact of exercise or NMN on body weight over time, separate repeated one-way ANOVAs were performed in the offspring of obese mothers that consumed chow (**B**) or HFD (**C**). A separate two-way ANOVA was performed to analyse the AUC in these animals (**E**). To explore the impact of interventions (exercise and NMN) on GTT in the offspring consuming chow (**F**) and HFD (**G**), separate repeated one-way ANOVAs were performed. The significant effects are: ^†^ *P* < 0.05, ^††^ *P* < 0.01, ^†††^ *P* < 0.001 overall maternal HFD effect. ^***^P < 0.001 overall post-weaning diet effect. ^X^P < 0.05, ^XX^P < 0.01, ^XXX^P < 0.001 simple main effect of exercise. ^^^P < 0.05, ^^^^^P < 0.001 simple main effect of NMN.

Maternal HFD had a significant impact on pup body weight (F(1,52) = 54.122, p < 0.001, Fig. 1A) whereby pups from mothers consuming HFD were heavier than those from lean mothers. The impact of maternal obesity was exacerbated if pups went on to HFD (F(1,52) = 11.914, p < 0.001) Fig. 1A. At 19 weeks the post-weaning HFD impact was greater in those from obese mothers (44% heavier) than lean mothers (15% heavier than controls) (Table 1).

**Table 1:** Body weight and organ mass in offspring of lean and obese mothers that consumed chow or HFD post-weaning

						Overall Effects		
		CCS	CHS	HCS	HHS	Maternal diet	Post Weaning Diet	Interaction
Final BW (g)		18.1 ± 0.2	20.9 ± 0.1^***^	19.5 ± 0.3	28.04 ± 1.2^†††/***^	F(1,52) = 62.33,P < 0.001	F(1,52) = 110.37, P < 0.001	F(1,52) = 27.24, P < 0.001
Liver (mg)		765 ± 14	693 ± 13^*^	779 ± 43	852 ± 27^†††^	F(1,48) = 11.9, P < 0.001	ns	F(1,48) = 8.4, P < 0.01
Muscle (mg)	Quad	236 ± 10	256 ± 11	247 ± 10	295 ± 8	F(1,50) = 5.7, P < 0.05		ns
	AT	82 ± 4	88 ± 3	87 ± 3	98 ± 5	F(1,52) = 4.2, P < 0.05	F(1,52) = 4.9, p < 0.05	ns
	Soleus	12.4 ± 0.7	15.9 ± 0.7	15.1 ± 0.7	19.6 ± 0.6	F(1,51) = 21.6, P < 0.001	F(1,51) = 34.7, p < 0.001	ns
WAT (mg)	Gonadal	141 ± 8	601 ± 89^***^	231 ± 22	1348 ± 164^†††/***^	F(1,51) = 22.3, P < 0.001	F(1,51) = 79.1, P < 0.001	F(1,51) = 13.7, P < 0.001
	Inguinal	165 ± 11	463 ± 57^***^	207 ± 13	823 ± 108^†††/***^	F(1,49) = 12.4, P < 0.001	F(1,49) = 63.7, P < 0.001	F(1,49) = 7.7, P < 0.01
	RP	26.1 ± 2.3	143.0 ± 25.2^***^	44.5 ± 4.2	347.5 ± 33.5^†††/***^	F(1,52) = 29.9, P < 0.001	F(1,52) = 106.5, P < 0.001	F(1,52) = 20.9, P < 0.001
Plasma Insulin (ng/mL)		0.085 ± 0.020	0.154 ± 0.016^***^	0.166 ± 0.020	0.420 ± 0.0.038^†††/***^	F(1,32) = 38.2, P < 0.001	F(1,32) = 33.2, P < 0.001	F(1,32) = 10.9, P < 0.01
Plasma Triglycerides (mg/mL)		2.82 ± 0.24	3.21 ± 0.16^**^	2.94 ± 0.11	3.62 ± 0.22^**^	ns	F(1,36) = 7.8, P < 0.01	ns

Data are shown as mean ± SEM, n = 11–16 per group. The first letter indicates maternal HFD, chow (**C**) or HFD (**H**); second letter indicates post-weaning diet (**C** or **H**); the third letter represents intervention; sedentary (**S**). To explore the impact of maternal and post-weaning diet on body weight and tissue phenotypes in the sedentary offspring groups CCS, CHS, HCS and HHS were analysed by two-way mixed ANOVA. Abbreviations: BW, body weight; WAT, white adipose tissue. Main effects are presented in the right hand panel. The significant effects are:

^†††^ *P* < 0.001 overall maternal HFD effect.

^*^ *P* < 0.05, ^***^ *P* < 0.001 overall post-weaning diet effect.

ns, no significant difference.

At 9 weeks of age chow or HFD fed offspring from HFD mothers were further distributed into sub groups: sedentary, exercise and NMN. Note, at this time-point NMN was not yet implemented. At 14 and 15 weeks of age exercise was associated with body weight reductions both in mice consuming chow (p < 0.05) and HFD (p < 0.05) (Fig. 1B and C). At 19 weeks NMN had reduced body weight in both the chow fed and HFD groups (P < 0.05) (Table 2).

**Table 2:** Body weight and organ mass in sedentary or exercised or NMN treated offspring of obese mothers that consumed chow or HFD post-weaning.

	CCS	CHS	HCS	HCX	HCN	HHS	HHX	HHN
Final BW (g)	18.1 ± 0.2	20.9 ± 0.1	19.5 ± 0.3	18.5 ± 0.3^X^	18.7 ± 0.2^	28.04 ± 1.2	24.3 ± 0.9^XX^	24.8 ± 0.7^
Liver (mg)	765 ± 14	693 ± 13	779 ± 43	758 ± 23	811 ± 18	852 ± 27	779 ± 26	763 ± 22
Muscle (mg)								
Quad	236 ± 10	256 ± 11	247 ± 10	254 ± 12	272 ± 6	295 ± 8	303 ± 5	304 ± 7
AT	82 ± 4	88 ± 3	87 ± 3	86 ± 3.21	87 ± 3	98 ± 5	110 ± 4	100 ± 5
Soleus	12.4 ± 0.7	15.9 ± 0.7	15.1 ± 0.7	15.0 ± 0.5	16.3 ± 1.1	19.6 ± 0.6	20.2 ± 0.5	19.2 ± 0.7
WAT (mg)								
Gonadal	141 ± 8	601 ± 89	231 ± 22	231 ± 14	202 ± 15	1348 ± 16	946 ± 149^X^	923 ± 92^
Inguinal	165 ± 11	463 ± 57	207 ± 13	232 ± 14	196 ± 5	823 ± 108	512 ± 66^XXX^	532 ± 58^^
RP	26.1 ± 2.3	143.0 ± 25.2	44.5 ± 4.2	43.1 ± 2.4	40.5 ± 2.2	347.5 ± 33.5	252.0 ± 38.2^XX^	220.5 ± 22.1^^^
Plasma Insulin (ng/mL)	0.085 ± 0.020	0.154 ± 0.016	0.166 ± 0.020	0.224 ± 0.037	0.317 ± 0.030^	0.420 ± 0.0.038	0.469 ± 0.033	0.588 ± 0.064^^
Plasma Triglycerides (mg/mL)	2.82 ± 0.24	3.21 ± 0.16	2.94 ± 0.11	3.38 ± 0.23	2.31 ± 0.41	3.62 ± 0.22	3.83 ± 0.24	3.14 ± 0.26
**Main effects of the ANOVA analysis of HC, HCX, HCN, HH, HHX, HHN offspring**								
		**Intervention**			**Post Weaning Diet**		**Interaction**	
Kill BW (g)		F(2,64) = 6.7, P < 0.01			F(1,64) = 135.5, P < 0.001		ns	
Liver (mg)		ns			ns		ns	
Muscle (mg)	Quad	ns			F(1,63) = 39.4, P < 0.001		ns	
	AT	ns			F(1,64) = 26.1, P < 0.001		ns	
	Soleus	ns			F(1,62) = 49.5, p < 0.001		ns	
WAT (mg)	Gonadal	F(1,65) = 3.1, P < 0.05			F(1,65) = 110.7, P < 0.001		ns	
	Inguinal	F(1,62) = 3.7, P < 0.05			F(1,62) = 74.9, P < 0.001		F(1,62) = 4.3, P < 0.05	
	RP	F(1,63) = 4.6, P < 0.05			F(1,63) = 157.3, P < 0.001		F(1,63) = 4.1, P < 0.05	
Plasma Insulin (ng/mL)		F(1,49) = 7.2, P < 0.01			F(1,49) = 54.7, P < 0.001		ns	
Plasma Triglycerides (mg/mL)		F(1,50) = 5.9, P < 0.01			F(1,50) = 9.6, P < 0.01		ns	

Data are shown as mean ± SEM, n = 11–12 per group. The first letter indicates maternal diet, chow (**C**) or HFD (**H**); second letter indicates post-weaning diet (**C** or **H**); the third letter represents intervention; sedentary (**S**), exercise (**X**) or NMN (**N**). To explore the impact of post-weaning diet and intervention (exercise and NMN) on body weight and tissue phenotypes, the offspring groups HCS, HCX, HCN and HHS, HHX, HHN were analysed by a two-way mixed ANOVA presented upper section of the table. Offspring from lean mothers (**CCS** & **CHS**) are shown for comparison. Abbreviations: BW, body weight; WAT, white adipose tissue. Overall effects are presented in the bottom section of the table. The significant effects are:

^X^P < 0.05, ^XX^P < 0.01, ^XXX^P < 0.001 simple main effect of exercise.

^^^P < 0.05, ^^^^P < 0.01, ^^^^^P < 0.001 simple main effect of NMN.

### Diet effects on organ mass of female offspring

To investigate impacts of maternal diet and post-weaning diet, various tissues were collected. Both maternal obesity and HFD consumption increased adiposity (Table 1). When tissue weight was standardised as % body weight, a significant maternal HFD effect was observed in liver, gonadal and retroperitoneal fat. A post-weaning diet effect was apparent in liver, quadricep and anterior tibialis muscles, gonadal, inguinal, and retroperitoneal fat, which were all increased (Table 2).

### Effect of post-weaning diet, exercise and NMN on tissue phenotypes

Mass of all three fat pads– gonadal, inguinal and retroperitoneal were significantly reduced by the interventions. No significant changes were induced in muscle or liver mass by the exercise and NMN interventions. A post-weaning HFD increased the mass of all three muscles and fat pads (Table 2), with no effect on liver weight. When the tissue weights were normalised to % body weight, a main effect of post weaning diet was observed in quadricep and AT muscle and all three fat pads as well as liver.

### Glucose tolerance and basal insulin

Both maternal (F(1,44) = 14.768, p < 0.001) and post-weaning (F(1,44) = 108.719, p < 0.001) diet had significant impacts on plasma glucose clearance suggesting that offspring from obese mothers were less glucose tolerant (Fig. 1D). As expected offspring consuming HFD were significantly glucose intolerant relative to those consuming chow, regardless of their mothers diet. The area under the GTT curve presented in Fig. 1E shows significant effects of maternal (F(1,48) = 18.8, p < 0.001) and post-weaning diet (F(1,48) = 115.7, p < 0.001).

No significant effect of intervention was observed in offspring consuming chow (Fig. 1F). In siblings consuming HFD, exercise and NMN improved the late phase of the response to glucose challenge (Fig. 1G). Measurement of the area under the curve (not shown) suggested that there are beneficial effects of both interventions. However only exercise reached significance (HHS vs HHEX P = 0.009; HHS vs HHN P = 0.059).

Both maternal and post-weaning diet had significant impacts on plasma insulin. Maternal and post weaning HFD increased plasma insulin concentration (Table 1). Furthermore, NMN significantly increased insulin concentration regardless of their diet (Table 2).

### NAD^+^ and NADH levels in liver

**Figure 2:**
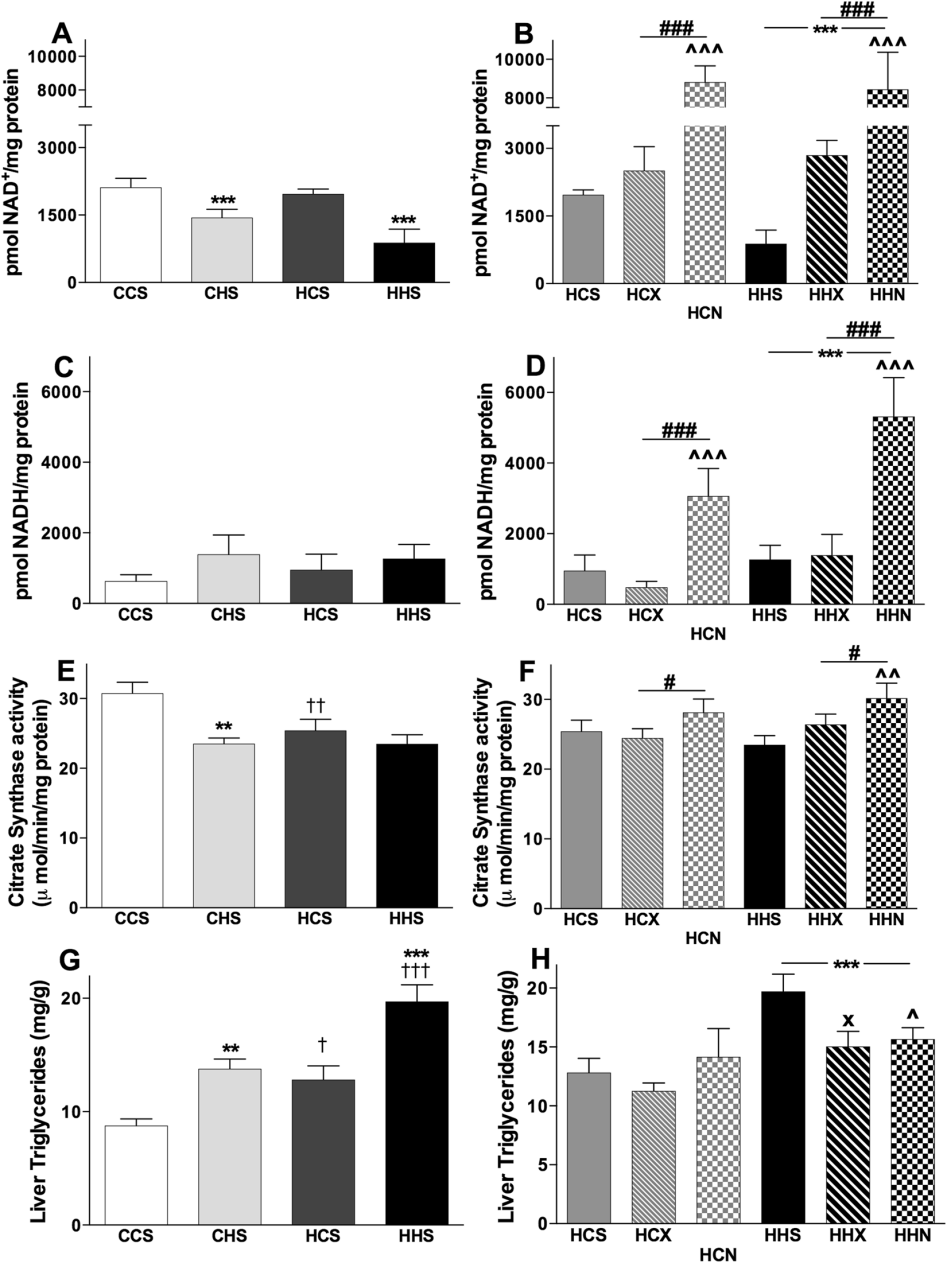
Effects of maternal diet, post-weaning diet, exercise and NMN on Hepatic NAD^+^ - NADH, citrate synthase activity and triglyceride content. Data are shown as mean ± SEM, n = 5–12 per group. The first letter represents maternal and second letter represents post-weaning diet; chow (**C**) or HFD (**H**); the third letter represents intervention; sedentary (**S**), exercise (**X**) or NMN (**N**). To investigate the effects of maternal and post-weaning diets on CCS, CHS, HCS and HHS groups on NAD^+^ (**A**), NADH (**C**), citrate synthase activity (**E**) and triglyceride (**G**) contents two-way ANOVAs were performed. To investigate the effects of post-weaning diet and intervention (exercise and NMN) on NAD^+^ (**B**), NADH (**D**) citrate synthase activity (**F**) and triglyceride (**H**) contents of the offspring from obese mothers consuming chow or HFD, HCS, HCX, HCN and HHS, HHX, HHN groups were compared by two-way ANOVA. The significant effects are: ^†^P < 0.05, ^††^P < 0.01 and ^†††^P < 0.001 overall maternal HFD effect. ^**^P < 0.01 and ^***^P < 0.001 overall post-weaning diet effect. ^#^P < 0.05 and ^###^P < 0.001 overall intervention effect. ^X^P < 0.05 simple main effect of exercise. ^^^P < 0.05, ^^^^P < 0.01 and ^^^^^P < 0.001 simple main effect of NMN.

While maternal diet had no effects, post-weaning HFD reduced NAD^+^ levels (F(1,16) = 17.599, p = 0.001) (Fig. 2A). There were no effects of post-weaning or maternal diet on concentrations of the reduced form, NADH (Fig. 2C). Interventions increased NAD^+^ concentrations in the offspring from obese mothers (F(1,24) = 36.306, p < 0.001, Fig. 2B). A significant increase in NADH concentrations was also seen with NMN treatment (F(1,24) = 15.681, p < 0.001, Fig. 2D), in line with the increased NAD^+^ concentrations. Exercise had no impact on NADH concentrations. Post-weaning HFD consumption was associated with significant increases in NADH (F(1,24) = 4.7, p < 0.05). Overall, there was a detrimental impact of post-weaning HFD on NAD^+^ concentrations, an increase with NMN treatment, with a more modest impact of exercise.

### Liver citrate synthase activity

Both maternal (F (1,34) = 3.9, p = 0.054) and post weaning (F(1,34) = 11.496, p < 0.01) diet reduced citrate synthase activity, but there was no additive effect (Fig. 2E). NMN treatment, but not exercise, increased citrate synthase activity in HFD consuming offspring (F(1,52) = 4.4, p < 0.01, Fig. 2F).

### Liver and plasma triglycerides

Liver triglyceride content shows an interesting graded response whereby both maternal (F (1,36) = 21.118, p < 0.001) and post-weaning (F(1,36) = 29.845, p < 0.001) HFD led to increased fat storage. The combination of maternal and post-weaning HFD led to an approximate doubling of hepatic triglyceride content compared to CCS (Fig. 2G). Offspring consuming HFD showed a significant reduction in hepatic triglyceride accumulation in response to both interventions; exercise and NMN (Fig. 2H).

Post-weaning HFD significantly increased plasma triglyceride with no maternal diet effect (Table 1). Neither intervention significantly altered plasma triglyceride concentration (Table 2).

### Maternal and post weaning diet affected expression of genes related to fat metabolism and gluconeogenesis

A range of genes involved in fat metabolism and gluconeogenesis were investigated for effects of maternal and post-weaning diet on their mRNA expression across CCS, CHS, HCS and HHS groups (Table 3). *Cd36*, a marker for fat transport into liver, showed higher mRNA expression in the liver in response to both maternal and post-weaning HFD. A significant effect of maternal HFD on *Pparg* mRNA expression, suggesting that maternal HFD was associated with increased fatty acid storage, where post-weaning HFD exacerbated the condition. Expression of *Fasn* mRNA was significantly decreased by post-weaning HFD consumption. Acetyl CoA carboxylase (*Acc1*) was higher in offspring from obese mothers and from lean mothers when offspring were consuming HFD. Mitochondrial pyruvate carrier *Mpc1* is responsible for transporting pyruvate out of the mitochondria for gluconeogenesis. Here, maternal and post-weaning HFD both increased *Mpc1* expression suggesting increased capacity for gluconeogenesis. Increases due to offspring HFD, but not maternal HFD were also seen in Pyruvate carboxylase (*PC*) and Phosphoenolpyruvate carboxykinase (*Pck1*) which are other genes that function in the early stages of gluconeogenesis. However, no effects were seen in the mRNA levels of the gene that is responsible for the terminal step of the process, Glucose-6-phosphatase (*G6pc*).

**Table 3:** mRNA expression of fat metabolism and gluconeogenesis markers in offspring of lean and obese mothers that consumed chow or HFD post-weaning.

Gene	CCS	CHS	HCS	HHS	Maternal diet	Post weaning diet	Interaction
*Acc1*	1.00 ± 0.05	1.28 ± 0.07^**^	1.27 ± 0.09^††^	1.16 ± 0.08	ns	ns	F (1, 34) = 7.522, p < 0.01
*Acc2*	1.00 ± 0.05	0.99 ± 0.04	0.97 ± 0.09	0.86 ± 0.10	ns	ns	ns
*Cd36*	1.00 ± 0.06	1.37 ± 0.08	1.48 ± 0.13	1.55 ± 0.16	F (1,33) = 8.982, p < 0.01	F(1,33) = 4.231, p < 0.05	ns
*Fasn*	1.00 ± 0.13	0.59 ± 0.05	0.89 ± 0.08	0.53 ± 0.03	ns	F(1,31) = 23.58, p < 0.001	ns
*Pgc1a*	1.00 ± 0.07	0.79 ± 0.06	0.96 ± 0.02	0.85 ± 0.06	ns	F(1,31) = 6.1, p < 0.05	ns
*Mpc1*	1.00 ± 0.07	1.45 ± 0.07	1.31 ± 0.08	1.53 ± 0.11	F (1,33) = 5.435, p < 0.05	F(1,31) = 16.285, p < 0.001	ns
*PC*	1.00 ± 0.07	1.32 ± 0.18	0.76 ± 0.08	0.97 ± 0.07	ns	F(1,33) = 6.051, p < 0.05	ns
*Pck1*	1.00 ± 0.22	1.49 ± 0.23	1.00 ± 0.19	1.27 ± 0.26	ns	F(1,31) = 4.710, p < 0.05	ns
*G6pc*	1.00 ± 0.14	1.09 ± 0.19	1.00 ± 0.18	0.99 ± 0.24	ns	ns	ns
*Pparg*	1.00 ± 0.12	0.56 ± 0.06^**^	1.82 ± 0.24^†††^	2.82 ± 0.48^†††/***^	F (1, 30) = 20.9, p < 0.001		F (1, 30) = 4.6, p < 0.05
*Sirt1*	1.00 ± 0.04	1.20 ± 0.03	0.89 ± 0.05	1.12 ± 0.09	ns	ns	ns
*Hadh*	1.00 ± 0.05	1.12 ± 0.06	1.10 ± 0.06	1.15 ± 0.07	ns	ns	ns
*Cpt1*	1.00 ± 0.06	1.01 ± 0.08	1.12 ± 0.10	1.00 ± 0.07	ns	ns	ns

Data are shown as mean ± SEM, n = 9–10 per group. The first letter indicates maternal diet, chow (**C**) or HFD (**H**); second letter indicates post-weaning diet (**C** or **H**); the third letter represents intervention; sedentary (**S**). To explore the impact of maternal and post-weaning diet on body weight and tissue phenotypes in the sedentary offspring groups CCS, CHS, HCS and HHS were analysed by two-way mixed ANOVA. Overall effects are presented in the right hand panel. Data are presented as fold changes compared to the CCS group. Abbreviations: Acc: Acetyl CoA Carboxylase; *Cd36*: Cluster of differentiation 36 / fatty acid translocase; *Fasn*: fatty acid synthase; *Pparg*: Peroxisome proliferator-activated receptor; *Mpc1*: mitochondrial pyruvate career*; Sirt1*: silent mating type information regulation; *PC*: Pyruvate carboxylase; *Pck1*: Phosphoenolpyruvate carboxykinase; *G6pc*: Glucose-6-phosphatase.

The significant effects are:

^††^ *P* < 0.01, ^†††^ *P* < 0.001 overall maternal HFD effect.

^**^P < 0.01, ^***^P < 0.001 overall post-weaning diet effect.

ns, no significant difference.

### Offspring diet and interventions affected expression of genes related to fat metabolism and gluconeogenesis

**Figure 3:**
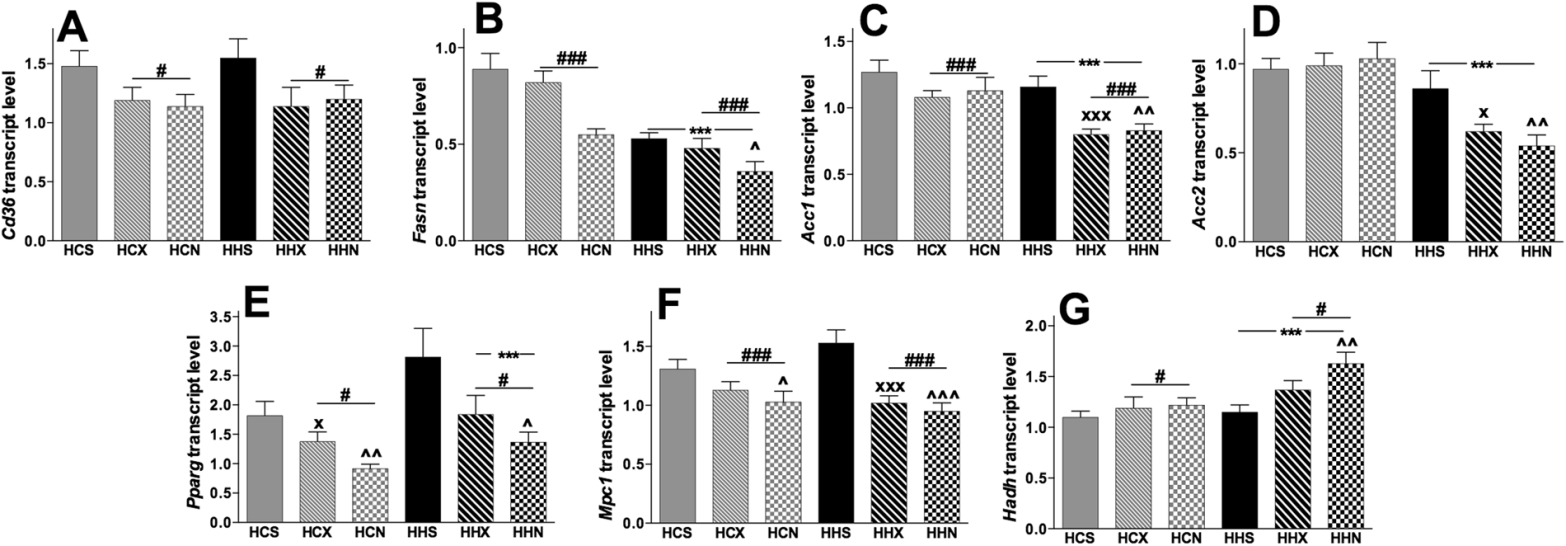
Effect of NMN and exercise on expression of genes involved in fat transport, synthesis and β-oxidation in liver. Data are shown as mean ± SEM, n = 9–10 per group. The first letter represents maternal and second letter represents post-weaning diet; chow (**C**) or HFD (**H**); the third letter represents intervention; sedentary (**S**), exercise (**X**) or NMN (**N**). Data are presented as fold changes compared to the CCS group. To investigate the effects of post-weaning diets and intervention (exercise and NMN) on mRNA expression of *Cd36* (**A**), *Fasn* (**B**), *Acc1* (**C**), *Acc2* (**D**), *Pparg* (**E**), *Mpc1* (**F**), *Hadh* (**G**) of the offspring of obese mothers consuming chow or HFD, HCS, HCX, HCN and HHS, HHX, HHN were compared by separate two-way ANOVAs. The significant effects are: ***P < 0.001 overall post-weaning diet effect. ^#^P < 0.01 and ^###^P < 0.001 overall intervention effect. ^X^P < 0.05 and ^XXX^P < 0.001 simple main effect of exercise. ^^^P < 0.05; ^^^^P < 0.01 and ^^^^^P < 0.01 simple main effect of NMN.

Gene expression in female offspring from obese mothers are depicted in Fig. 3. The effect of maternal HFD to increase *Cd36* and *Fasn* seen in Table 3 was reduced by both interventions (Fig. 3A,B). NMN reduced the mRNA expression of *Fasn* significantly, compared to vehicle control in both diets. A significant effect of post-weaning diet (F(1,51) = 36.9, p < 0.001) suggests that there is also a HFD effect to decrease expression of *Fasn* (Fig. 3B). There are two subunits of acetyl-CoA carboxylase, *Acc1* and *Acc2* (responsible for fatty acid synthesis and production of malonyl CoA). Both exercise (P < 0.001) and NMN (P < 0.01) reduced *Acc1* expression only when pups consumed HFD (Fig. 3C). Both interventions exerted similar effects on *Acc2* gene expression (Fig. 3D). *Mpc1* (Fig. 3E) and *Pparg* (Fig. 3F) mRNA expression were significantly reduced by NMN treatment in mice from obese mother consuming either chow or HFD. A significant increase of *Hadh* mRNA was induced by NMN only when offspring consumed HFD post-weaning (Fig. 3G). Analysis revealed a significant effect of post-weaning diet on *Hadh* mRNA expression, F(1,52) = 15.086, p < 0.001), which suggests a possible increase in β-oxidation in liver. However, there were no changes observed in *Cpt1* mRNA expression (Supplementary Fig. S3). The effect of the interventions on liver gluconeogenesis was less clear than on fat metabolism in that organ. Both NMN and exercise significantly reduced *Mpc1* transcript levels which may suggest reduced gluconeogenesis (Supplementary Fig. S3). However, no significant intervention effects were seen in other markers.

No changes in hepatic *Sirt1* mRNA expression were observed in response to post-weaning diet or either intervention. We found significant changes in *Pgc1α* mRNA expression, whereby post weaning diet reduced expression (F(1,53) = 36.7, p < 0.001). Exercise increased *Pgc1α* expression significantly only when pups consumed chow diet (Supplementary Fig. S3C). There was no maternal diet effect; however post-weaning HFD increased the mtDNA copy number with no significant effect of interventions.

## Discussion

The correlations between maternal body weight during pregnancy and long-term offspring health risks mean that there is much interest in finding effective interventions to reduce these risks. Studies in humans and animals have shown that strategies that increase energy utilisation either through pharmacological approaches or physical activity can be effective. Here we wanted to test whether physical exercise or NMN treatment in offspring of obese mothers would improve metabolism, as we have previously shown in female mice exposed to diet induced obesity^26^.

In this study, as expected, the HFD induced greater accumulation of adipose tissue in the mothers, and a subsequent increase in pup body weight prior to weaning. A feature of programmed phenotypes is that they are often latent until exposed by a second environmental factor that occurs later in life. A clear illustration of this is the detection or exacerbation of phenotypes due to the combined impact of maternal obesity and offspring eating HFD themselves^39–41^. In this study significant differences were observed between mice that experienced the combined metabolic insult of maternal and offspring HFD (i.e. HHS) and the group that experienced post-weaning HFD alone (CHS). These differences were in final body weight, organ weights, GTT, plasma insulin, liver triglyceride and Pparg transcript levels. In addition, comparing CCS and HCS groups revealed some programmed phenotypes even without the exacerbation of post-weaning HFD, specifically in liver triglyceride, CS activity and *Mpc1*, *Pparg* and *Cd36* transcript. In all instances these programmed changes (either latent or manifest) were in the same direction as those caused by post-weaning HFD alone (CCS vs CHS) which suggests that there is a high similarity in the regulatory and physiological effects of maternal obesity and diet induced obesity on fat metabolism and markers of mitochondrial function in the liver. These observations are consistent with previous studies that investigated programming by maternal obesity^21,41–43^. To our knowledge we are the first to investigate how maternal obesity affects NAD^+^ concentrations. Our data support earlier reports^26,27^, of reduced NAD^+^ concentrations in response to post-weaning HFD consumption. There also appeared to be a slightly greater (non-significant) reduction in those from obese mothers, which may suggest that liver NAD^+^ metabolism is influenced by programmed processes such as those that also reduced CS activity. Further work is needed to understand how i.p. NMN injection leads to increased NAD^+^ levels in the liver. Previous work has shown that increases in liver NAD^+^ can be detected 30 minutes after oral administration of NMN^44^. However, the relative importance of the NAD^+^ biosynthetic and salvage pathways^45^ in both NMN supplementation and exercise requires more investigation.

Effects of NMN supplementation on hepatic fat metabolism were considerable. High levels of NAD^+^ are known to increase mitochondrial function and biogenesis^46^. In this study NMN led to marked increases in hepatic NAD^+^ levels, with similar impacts in offspring consuming chow or HFD. High NAD^+^ concentrations in liver following NMN supplementation may reflect the pharmacokinetics related to i.p. injection. Moreover, NMN was injected on the morning of tissue harvest.

Most intervention studies using the NAD^+^-precursor NR or the allosteric sirtuin activator resveratrol^47^ in HFD-induced obesity in mice have reported reduced body weight and fat accumulation^48–50^. Unlike previous studies where no weight changes were observed in HFD fed mice with 7^27^ or 10^26^ days of i.p. NMN treatment^27^, here for the first time we found a significant reduction in final body weight with a slightly longer period of NMN (18 days). Reductions in fat mass were observed in response to both exercise and NMN; these were more pronounced in offspring consuming HFD, and they were of similar magnitude across both interventions. While it appeared NMN may have led to some fat loss in offspring consuming chow, this failed to reach significance.

Our findings showed increased triglyceride accumulation in response to both maternal and post weaning HFD. This is an indicator of major metabolic impairment. NMN supplementation partially reversed liver triglyceride accumulation. Lower hepatic triglyceride levels can be related to several processes: increased fat catabolism, reduced fat synthesis, or reduced fat import into the liver. Our results suggest that all three processes are altered by NMN in ways that would reduce liver fat content. The increases in mitochondrial activity (CS activity) and β-oxidation (*Hadh* levels) would increase fat catabolism. Decreases were also seen in genes involved in fat synthesis (*Fasn*, *Acaca*, *Acacb*) and storage (*Pparg*). Finally, reduced *Cd36* suggests that the NMN-supplemented liver is importing less fatty acid.

In HHS groups the reduction in liver triglyceride was equivalent in the exercise (HHX) or NMN (HHN) groups. However, it is likely that there are mechanistic differences in the ways these interventions reduce liver triglyceride. As mentioned above NMN-supplementation led to large increases in liver NAD^+^ while exercise did not. This excess of NAD^+^ is expected to increase a variety of sirtuin mediated processes such as mitochondrial function, fatty acid synthesis and β-oxidation^49,51,52^. In support of this we found that NMN altered CS activity and *FASN*, *Hadh* and *Pgc-1α*, transcript levels but exercise did not. However, direct measurements of fatty acid oxidation or sirtuin activity would answer this more precisely. It should also be noted that as animals were sacrificed 18–21 h post exercise, it is possible that some measurements (e.g. mRNA levels) represent a lingering effect of the previous exercise session, rather than those of chronic exercise.

As expected, maternal and post-weaning HFD induced glucose intolerance and increased plasma insulin levels. NMN and exercise both partially improved glucose tolerance in the post-weaning HFD groups. The improvement of glucose tolerance in exercise is most likely due to improved insulin sensitivity and glucose uptake, especially by skeletal muscle^53^. In contrast, our data suggest that NMN supplementation may increase clearance of glucose from the circulation by increasing plasma insulin levels. It is unclear whether the heightened plasma insulin in our cohort is explained by *Sirt1*-mediated increases in pancreatic islet beta cell mass^54^ or by increased *Sirt1*-mediated insulin secretion, as NMN was administered 4 hours prior to sacrifice.

We found that 18 days of NMN supplementation had similar beneficial effects to 9 weeks of exercise in a mouse model of maternal obesity. The two interventions lowered body weights to the same degree (Table 2) thus the differential effects cannot be attributed to indirect influence of body weight. Few studies to date have examined longer term NMN treatment; in a mouse model of aging 12 months NMN treatment improved a variety of age-associated phenotypes including energy metabolism, eye function, bone density and immune function with no evidence of toxicity^44^. We are currently investigating the impact of longer term NMN treatments in the context of diet-induced and maternal obesity.

Our results also suggest that improved fat metabolism in liver due to post-weaning NMN supplementation and exercise are significant contributors to the overall improvements in adiposity. The interventions appeared to exert the most benefits in the offspring that were most metabolically challenged, i.e. the HFD consuming offspring of obese females. This supports the suitability of NMN for use in reversing metabolic dysfunction linked to programming, as well those induced by an individual’s lifestyle. Mechanistically, there are significant differences in the changes induced by exercise and NMN supplementation, which may be due to the latter increasing NAD^+^ concentrations in liver and consequently stimulating sirtuin activity.

## Electronic supplementary material

Supplementary Information

## References

[1] Ng M (2014). Global, regional, and national prevalence of overweight and obesity in children and adults during 1980–2013: a systematic analysis for the Global Burden of Disease Study 2013. The Lancet.

[2] Mitchell S, Shaw D (2015). The worldwide epidemic of female obesity. Best Practice & Research Clinical Obstetrics & Gynaecology.

[3] Lu GC (2001). The effect of the increasing prevalence of maternal obesity on perinatal morbidity. American journal of obstetrics and gynecology.

[4] Castillo-Laura H, Santos IS, Quadros LC, Matijasevich A (2015). Maternal obesity and offspring body composition by indirect methods: a systematic review and meta-analysis. Cadernos de saude publica.

[5] Yu Z (2013). Pre-pregnancy body mass index in relation to infant birth weight and offspring overweight/obesity: a systematic review and meta-analysis. PloS one.

[6] Morris MJ (2009). Early life influences on obesity risk: maternal overnutrition and programming of obesity. Expert Review of Endocrinology & Metabolism.

[7] Nicholas LM (2016). The early origins of obesity and insulin resistance: timing, programming and mechanisms. Int J Obes (Lond).

[8] Li, M., Sloboda, D. & Vickers, M. Maternal obesity and developmental programming of metabolic disorders in offspring: evidence from animal models. *Experimental diabetes research* 2011 (2011). 10.1155/2011/592408 PMC3182397 21969822

[9] Boney CM, Verma A, Tucker R, Vohr BR (2005). Metabolic syndrome in childhood: association with birth weight, maternal obesity, and gestational diabetes mellitus. Pediatrics.

[10] Must, A. & Strauss, R. S. Risks and consequences of childhood and adolescent obesity. International Journal of Obesity & Related Metabolic Disorders **23** (1999). 10.1038/sj.ijo.0800852 10340798

[11] Muhlhausler BS, Gugusheff JR, Ong ZY, Vithayathil MA (2013). Nutritional approaches to breaking the intergenerational cycle of obesity. Canadian journal of physiology and pharmacology.

[12] Wofford LG (2008). Systematic review of childhood obesity prevention. Journal of Pediatric Nursing.

[13] Hopkins SA, Baldi JC, Cutfield WS, McCowan L, Hofman PL (2010). Exercise training in pregnancy reduces offspring size without changes in maternal insulin sensitivity. The Journal of Clinical Endocrinology & Metabolism.

[14] Raipuria M, Bahari H, Morris MJ (2015). Effects of maternal diet and exercise during pregnancy on glucose metabolism in skeletal muscle and fat of weanling rats. PloS one.

[15] Vega, C. C. *et al*. Exercise in obese female rats has beneficial effects on maternal and male and female offspring metabolism. *International Journal of Obesity* (2013). 10.1038/ijo.2013.150 PMC3925765 23949616

[16] Stanford KI (2015). Exercise before and during pregnancy prevents the deleterious effects of maternal high-fat feeding on metabolic health of male offspring. Diabetes.

[17] Sheldon RD (2016). Gestational exercise protects adult male offspring from high-fat diet-induced hepatic steatosis. Journal of hepatology.

[18] Segovia SA, Vickers MH, Zhang XD, Gray C, Reynolds CM (2015). Maternal supplementation with conjugated linoleic acid in the setting of diet-induced obesity normalises the inflammatory phenotype in mothers and reverses metabolic dysfunction and impaired insulin sensitivity in offspring. The Journal of nutritional biochemistry.

[19] Thomas H, Berlanga A, Byrne C, Cagampang F (2015). Maternal Metformin Treatment During Obese Pregnancy Reduces Offspring Fatty Liver and Hepatic Inflammation. The FASEB Journal.

[20] Vega, C. C. *et al*. Resveratrol partially prevents oxidative stress and metabolic dysfunction in pregnant rats fed a low protein diet and their offspring. *The Journal of physiology* (2016). 10.1113/JP271543 PMC4771783 26662841

[21] Bahari H, Caruso V, Morris MJ (2013). Late-onset exercise in female rat offspring ameliorates the detrimental metabolic impact of maternal obesity. Endocrinology.

[22] Agil A (2015). Melatonin reduces hepatic mitochondrial dysfunction in diabetic obese rats. J Pineal Res.

[23] Jheng H-F (2012). Mitochondrial fission contributes to mitochondrial dysfunction and insulin resistance in skeletal muscle. Molecular and cellular biology.

[24] Kim J-A, Wei Y, Sowers JR (2008). Role of mitochondrial dysfunction in insulin resistance. Circulation research.

[25] Mantena SK, King AL, Andringa KK, Eccleston HB, Bailey SM (2008). Mitochondrial dysfunction and oxidative stress in the pathogenesis of alcohol-and obesity-induced fatty liver diseases. Free Radical Biology and Medicine.

[26] Uddin GM, Youngson NA, Sinclair DA, Morris MJ (2016). Head to Head Comparison of Short-Term Treatment with the NAD(+) Precursor Nicotinamide Mononucleotide (NMN) and 6 Weeks of Exercise in Obese Female Mice. Front Pharmacol.

[27] Yoshino J, Mills KF, Yoon MJ, Imai S-I (2011). Nicotinamide mononucleotide, a key NAD+ intermediate, treats the pathophysiology of diet-and age-induced diabetes in mice. Cell metabolism.

[28] Cantó, C. & Auwerx, J. In *Cold Spring Harbor symposia on quantitative biology*. 291–298 (Cold Spring Harbor Laboratory Press). 10.1101/sqb.2012.76.010439 PMC3616234 22345172

[29] Massudi, H., Wu, L. E. & Sinclair, D. A. In *Sirtuins* 243–266 (Springer, 2016).

[30] Cantó C (2010). Interdependence of AMPK and SIRT1 for metabolic adaptation to fasting and exercise in skeletal muscle. Cell metabolism.

[31] Koltai E (2010). Exercise alters SIRT1, SIRT6, NAD and NAMPT levels in skeletal muscle of aged rats. Mechanisms of ageing and development.

[32] Geng T (2010). PGC-1α plays a functional role in exercise-induced mitochondrial biogenesis and angiogenesis but not fiber-type transformation in mouse skeletal muscle. American Journal of Physiology-Cell Physiology.

[33] Gomes AP (2013). Declining NAD+induces a pseudohypoxic state disrupting nuclear-mitochondrial communication during aging. Cell.

[34] Price NL (2012). SIRT1 is required for AMPK activation and the beneficial effects of resveratrol on mitochondrial function. Cell metabolism.

[35] Li J (2017). A conserved NAD+binding pocket that regulates protein-protein interactions during aging. Science.

[36] Igosheva N (2010). Maternal diet-induced obesity alters mitochondrial activity and redox status in mouse oocytes and zygotes. PLoS One.

[37] Wu LL (2015). Mitochondrial dysfunction in oocytes of obese mothers: transmission to offspring and reversal by pharmacological endoplasmic reticulum stress inhibitors. Development.

[38] Zhu C-T, Rand DM (2012). A hydrazine coupled cycling assay validates the decrease in redox ratio under starvation in Drosophila. PloS one.

[39] Chen H, Simar D, Morris MJ (2009). Hypothalamic neuroendocrine circuitry is programmed by maternal obesity: interaction with postnatal nutritional environment. PloS one.

[40] Rajia S, Chen H, Morris M (2010). Maternal Overnutrition Impacts Offspring Adiposity and Brain Appetite Markers‐Modulation by Postweaning Diet. Journal of neuroendocrinology.

[41] Chen H, Simar D, Lambert K, Mercier J, Morris MJ (2008). Maternal and postnatal overnutrition differentially impact appetite regulators and fuel metabolism. Endocrinology.

[42] Howie G, Sloboda D, Kamal T, Vickers M (2009). Maternal nutritional history predicts obesity in adult offspring independent of postnatal diet. The Journal of physiology.

[43] Alfaradhi MZ (2014). Oxidative stress and altered lipid homeostasis in the programming of offspring fatty liver by maternal obesity. American journal of physiology. Regulatory, integrative and comparative physiology.

[44] Mills KF (2016). Long-Term Administration of Nicotinamide Mononucleotide Mitigates Age-Associated Physiological Decline in Mice. Cell Metab.

[45] Imai S, Guarente L (2014). NAD+and sirtuins in aging and disease. Trends Cell Biol.

[46] Mouchiroud L, Houtkooper RH, Auwerx J (2013). NAD+ metabolism: a therapeutic target for age-related metabolic disease. Critical reviews in biochemistry and molecular biology.

[47] Hubbard BP (2013). Evidence for a common mechanism of SIRT1 regulation by allosteric activators. Science.

[48] Gariani, K. *et al*. Eliciting the mitochondrial unfolded protein response by nicotinamide adenine dinucleotide repletion reverses fatty liver disease in mice. *Hepatology* (2015). 10.1002/hep.28245 PMC4805450 26404765

[49] Cantó C (2012). The NAD+ precursor nicotinamide riboside enhances oxidative metabolism and protects against high-fat diet-induced obesity. Cell metabolism.

[50] Baur JA (2006). Resveratrol improves health and survival of mice on a high-calorie diet. Nature.

[51] Gariani K (2017). Inhibiting poly ADP-ribosylation increases fatty acid oxidation and protects against fatty liver disease. J Hepatol.

[52] Gariani, K. *et al*. Eliciting the mitochondrial unfolded protein response via NAD repletion reverses fatty liver disease. *Hepatology*, 10.1002/hep.28245 (2015). 10.1002/hep.28245 PMC4805450 26404765

[53] Christ CY (2002). Exercise training improves muscle insulin resistance but not insulin receptor signaling in obese Zucker rats. Journal of Applied Physiology.

[54] Gilbert RE (2015). SIRT1 activation ameliorates hyperglycaemia by inducing a torpor-like state in an obese mouse model of type 2 diabetes. Diabetologia.

