# Supplementary material for: Nicotinamide mononucleotide (NMN) supplementation ameliorates the impact of maternal obesity in mice: comparison with exercise
Source: Sci Rep. 2017 Nov 8;7:15063. doi: 10.1038/s41598-017-14866-z (PMC5678092; doi:10.1038/s41598-017-14866-z)
Supplement: Supplementary file 1 — Supplementary Information [file 41598_2017_14866_MOESM1_ESM.pdf]

Nicotinamide mononucleotide (NMN) supplementation ameliorates the impact of maternal obesity in mice: comparison with exercise

Golam Mezbah Uddin[1], Neil A. Youngson[1], Bronte M. Doyle[1] David A. Sinclair[1, 2], Margaret J. Morris[1*]

[1]Department of Pharmacology, School of Medical Sciences, UNSW, Sydney, NSW-2032, Australia

[2]Department of Genetics, Paul F. Glenn Laboratories for the Biological Mechanisms of Aging, Harvard Medical School Boston MA-02115, United States

*Corresponding author: Email: m.morris@unsw.edu.au

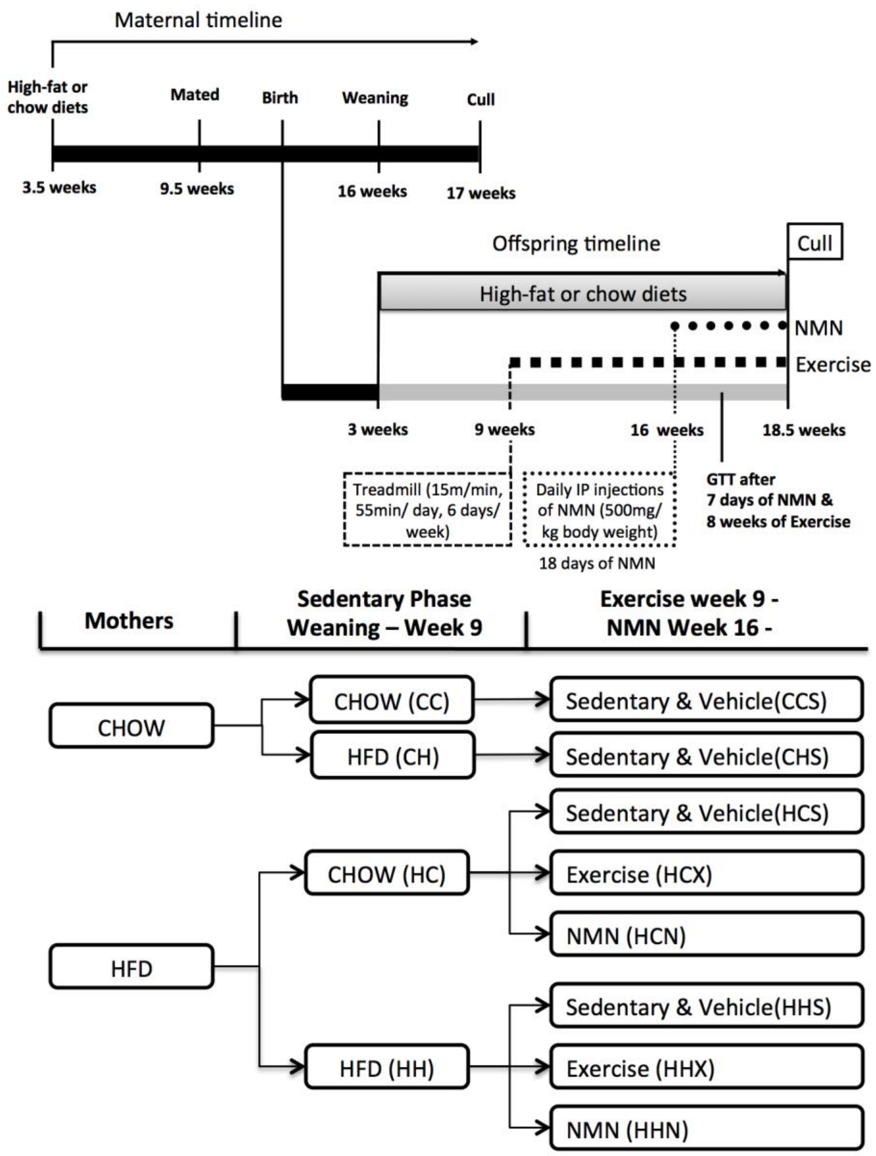

Supplementary Figure S1: Experimental Timeline. Top part shows the detail timeline of the experiment and bottom section representing the group distribution. First letter indicates maternal diet from 5 weeks prior to mating until weaning, chow (C) or HFD (H); second letter indicates offspring post-weaning diet (C or H); third letter indicates type of intervention – sedentary (S), treadmill exercise (X) or daily NMN injection (N).

	CHOW	HFD	Significance
Final Body Weight (g)	22.8±0.5	28.7±0.5	***
Glucose mM	6.3±0.1	7.6±0.1	***
Liver (mg)	778.6±23.5	947.0±14.2	
Heart (mg)	111.1±2.3	126.6±0.8	***
Quadriceps (mg)	241.2±1.4	286.0±1.5	***
Gonadal WAT (mg)	126.6±12.8	815.9±38.8	***
Liver % BW	3.4±0.1	3.3±0.1	
Heart % BW	0.49±0.01	0.44±0.01	
Quadriceps % BW	1.05±0.01	1.00±0.01	
Gonadal WAT % BW	0.6±0.1	2.8±0.1	***

Supplementary Table S2 Final body weight and dissected organ weights at 17 weeks of age.

Dissected tissue weight, as net mass and % body weight and basal glucose concentration of chow and HFD fed mothers. Data are shown as mean±SEM; n = 8 for organ mass data; 9–11 for body weight and blood glucose. Data were analysed using Student's t-test. Significant differences are presented as

*** P<0.001 diet effect

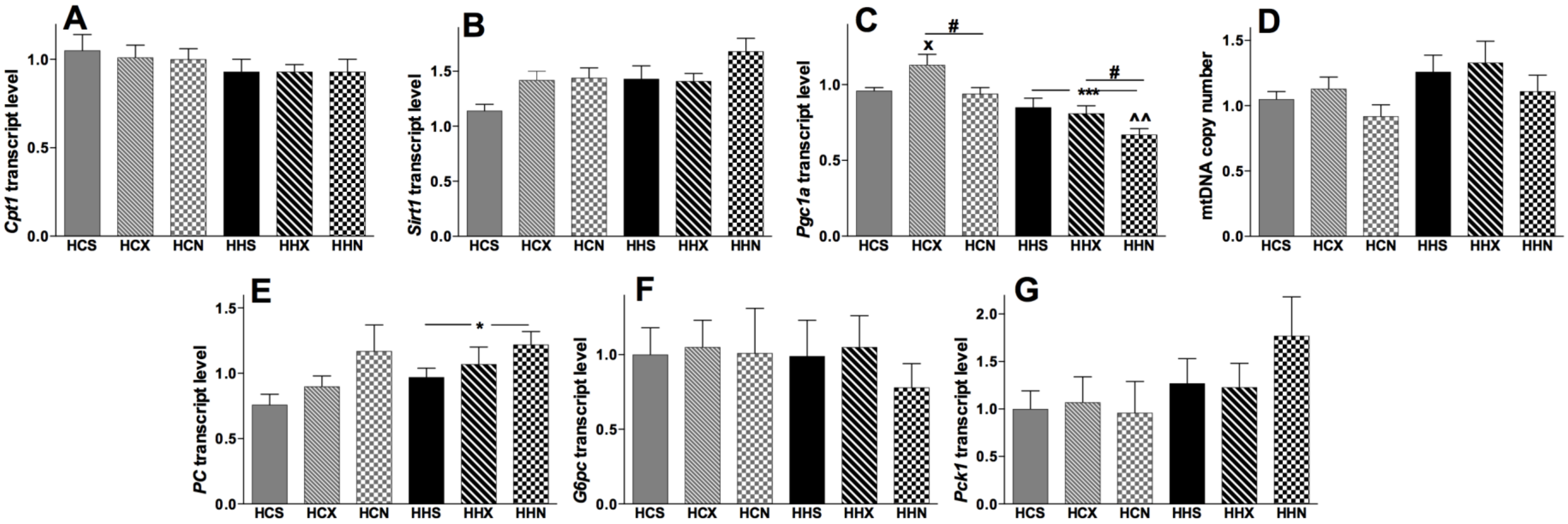

Supplementary Figure S3: Effect of NMN and exercise on mitochondrial DNA copy number and expression of genes involved in mitochondrial biogenesis, fat transport and gluconeogenesis in liver. Data are shown as mean±SEM, n = 9-10 per group. Data are presented as fold changes compared to the CCS group. The first letter represents maternal and second letter represents post-weaning diet; chow (C) or HFD (H); the third letter represents intervention; sedentary (S), exercise (X) or NMN (N). To investigate the effects of post-weaning diets and intervention (exercise and NMN) on mRNA expression of Cpt1 (**A**), Sirt1 (**B**), Pgc1a (**C**), mtDNA **(D)**, PC (**E**), G6pc (**F**) and Pck1 (**G**) of the offspring of obese mothers consuming chow or HFD, HC, HCX, HCN and HH, HHX, HHN were compared by separate two-way ANOVAs. The significant effects are:

*P<0.05, ***P<0.001 overall post-weaning diet effect

$^{\#}$ P<0.01 overall intervention effect

^{X} P<0.05 simple main effect of exercise

$^{\wedge\wedge}$ P<0.01 simple main effect of NMN

Gene	Forward	Reverse
Sirt 1	TGTAAGTTACTGCAGGAGTGTAAA	GCATAGATACCGTCTCTTGATCTGAA
Pgc1a	TATGGAGTGACATAGAGTGTGCT	CCACTTCAATCCACCCAGAAAG
Pparg	TCGCTGATGCACTGCCTATG	GAGAGGTCCACAGAGCTGATT
Fasn	AAGCCGTTGGGAGTGAAAGT	CAATCTGGATGGCAGTGAGG
Cd36	TGGCCTTACTTGGGATTGG	CCAGTGTATATGTAGGCTCATCCA
MPC1	GTGCTGCAGCCTTTCTCAC	GTTCCCACAAAGGCATCAC
Acc1	GCCTCCGTCAGCTCAGATAC	ATTCTGGCTGGAGAAGCCACA
Acc2	CATACACAGAGCTGGTGTTGGACT	CACCATGCCCACCTCGTTAC
Cpt1a	CAGAGGATGGACACTGTAAAGG	CGGCACTTCTTGATCAAGCC
Hadh	TTGCGCTCCATGTCCTCCTC	GACTCTCCTCAATTCCCTTC
Gapdh	AGGTCGGTGTGAACGGATTTG	TGTAGACCATGTAGTTGAGGT
Ywhaz	GAAAATGAAGGGTGACTACTAC	CTGATTTCAAATGCTTCTTG
Pck1	GGTATTGAACTGACAGACTC	CCAGTTGTTGACCAAAGG
G6pc	GTCGTGGCTGGAGTCTTG	CGGAGGCTGGCATTGTAG

Supplementary Table S4: Primer sequences of all target genes for RT-PCR
